# An Optimized Protocol for SBEM-Based Ultrastructural Analysis of Cultured Human Cells

**DOI:** 10.3390/mps8040090

**Published:** 2025-08-06

**Authors:** Natalia Diak, Łukasz Chajec, Agnieszka Fus-Kujawa, Karolina Bajdak-Rusinek

**Affiliations:** 1 Department of Molecular Biology, Faculty of Medical Sciences in Katowice, Medical University of Silesia, Medykow 18 Street, 40-752 Katowice, Poland; afus@sum.edu.pl (A.F.-K.); kbajdak-rusinek@sum.edu.pl (K.B.-R.); 2 Institute of Biology, Biotechnology and Environmental Protection, Faculty of Natural Sciences, University of Silesia in Katowice, Bankowa 9, 40-007 Katowice, Poland; lukasz.chajec@us.edu.pl

**Keywords:** SBEM protocols, iPSC, fibroblast, ultrastructure of cells

## Abstract

Serial block-face scanning electron microscopy (SBEM) is a powerful technique for three-dimensional ultrastructural analysis of biological samples, though its application to in vitro cultured human cells remains underutilized. In this study, we present an optimized SBEM sample preparation protocol using human dermal fibroblasts and induced pluripotent stem cells (iPSCs). The method includes key modifications to the original protocol, such as using only glutaraldehyde for fixation and substituting the toxic cacodylate buffer with a less hazardous phosphate buffer. These adaptations result in excellent preservation of cellular ultrastructure, with high contrast and clarity, as validated by transmission electron microscopy (TEM). The loss of natural cell morphology resulted from fixation during passage, when cells formed a precipitate, rather than from fixation directly within the culture medium. The protocol is time-efficient, safe, and broadly applicable to both stem cells and differentiated cells cultured under 2D conditions, providing a valuable tool for ultrastructural analysis in diverse biomedical research settings.

## 1. Introduction

Three-dimensional reconstructions are becoming an increasingly popular approach in various scientific disciplines, including biological and medical research. A wide array of techniques are available for reconstructing tissues and cells, such as confocal microscopy [1], array tomography [2], transmission electron microscopy (TEM) [3], serial block face scanning electron microscopy (SBEM) [4,5,6], scanning electron microscopy (SEM) [3], scanning transmission electron microscopy (STEM) [3], correlative light and electron microscopy (CLEM) [7], and micro-computed tomography (microCT) [8]. One of the standard and widely used methods in medical histopathology for preparing 3D tissue models is based on paraffin sectioning—for example, in bladder tumors reconstruction [9] or lung adenocarcinoma [10]. In the classical method, light microscopes are used to analyze specimens at the structural level.

For ultrastructural analysis, SBEM is a powerful technique. The concept of serial block-face imaging was first introduced in the 1980s [11], and later, the method was refined and described in detail by Denk and Horstmann in 2004 [12], who applied it to muscle and brain tissue reconstructions. SBEM generates results similar to TEM (2D), but it employs a scanning electron microscope equipped with an ultramicrotome. The operating principle of this microscope involves scanning the surface of a resin block (containing the embedded material), after which an internal knife slices off a thin section to expose a deeper layer, which is then scanned. This procedure is repeated multiple times to create a set of images, which can then be computationally reconstructed into a 3D model [12,13]. The quality and resolution of the final 3D reconstruction depend heavily on the sample preparation process, particularly on the fixation protocol used to preserve ultrastructural details.

A protocol for preparing biological specimens for SBEM was described by Deerinck et al. in 2010 [14]. This protocol remains widely used today, often with minor modifications tailored to specific tissues or experimental goals. These adjustments typically involve variations in buffer composition or the series of alcohols used for dehydration. Numerous adaptations of this protocol have been reported, for instance, for mouse hippocampal tissue [4] or cultured Vero cells [5,6]. Despite these advances, there is still a need to optimize fixation protocols for different cell types and experimental contexts, especially for in vitro applications.

In these studies, we evaluated and optimized a fixation protocol for SBEM in terms of ultrastructure analysis of cultured cells. Conventional TEM was employed to assess fixation quality. Unlike most studies that analyze cells derived from tissue slices, we used in vitro cultured cells: human skin fibroblasts (control) and induced pluripotent stem cells (iPSCs), both maintained in 2D culture conditions.

## 2. Materials and Methods

Equipment for Cell Culture:

- Primary Dermal Fibroblast; Human, Neonatal - ATCC, Virginia, USA, Cat. No. PCS-201-010™;
- Essential 8™ Medium—Life Technologies, Waltham, MA, USA 02451, Cat No. A1517001;
- DPBS no calcium, no magnesium—Life Technologies, Waltham, MA, USA 02451, Cat No. 14190144;
- UltraPure™—0.5M EDTA, pH 8.0, Life Technologies, Waltham, MA, USA 02451, Cat No. 15575020.

Equipment for SBEM Fixation:

- Glutaraldehyde—Sigma-Aldrich, Taufkirchen, Germany, Cat. No. G5882;
- Potassium hexacyanoferrate (III)—Warchem, Poland, Cat. No. 52731;
- Osmium tetroxide—Serva Cat. Heidelberg, Germany, No. 31251.03;
- Thiocarbohydrazide—Sigma-Aldrich, Taufkirchen, Germany, Cat. No. 223220;
- A 1% aqueous uranyl—SPI supplies, West Chester, USA Cat. No. 02624-AB;
- Lead nitrate—Supelco Taufkirchen, Germany, Cat. No. 1.07398;
- L- aspartic acid—Sigma-Aldrich, Taufkirchen, Germany, Cat. No. A9256;
- di-Sodium hydrogen phosphate dodecahydrate pure p.a.—Alchem, Poland, Cat. No. 363-117992809;
- Sodium dihydrogen phosphate monohydrate pure p.a.—Alchem, Poland, Cat. No. 363-117991804;
- Epoxy Embedding Medium—Polysciences, Hirschberg an der Bergstrasse, Germany Cat. No. 08792.

## 3. Procedure

**Cell culture**

Human skin fibroblasts (control) and induced pluripotent stem cells (iPSCs) were seeded at a density of 1 × 10^6^ cells for each 100 mm Petri dish. When 80% of confluency was reached, the cells were detached using 0.5 M EDTA (pH = 8.0) and centrifuged at 1000 RPM (run per minute) for 5 min in order to obtain a cell pellet.

**Figure 1:**
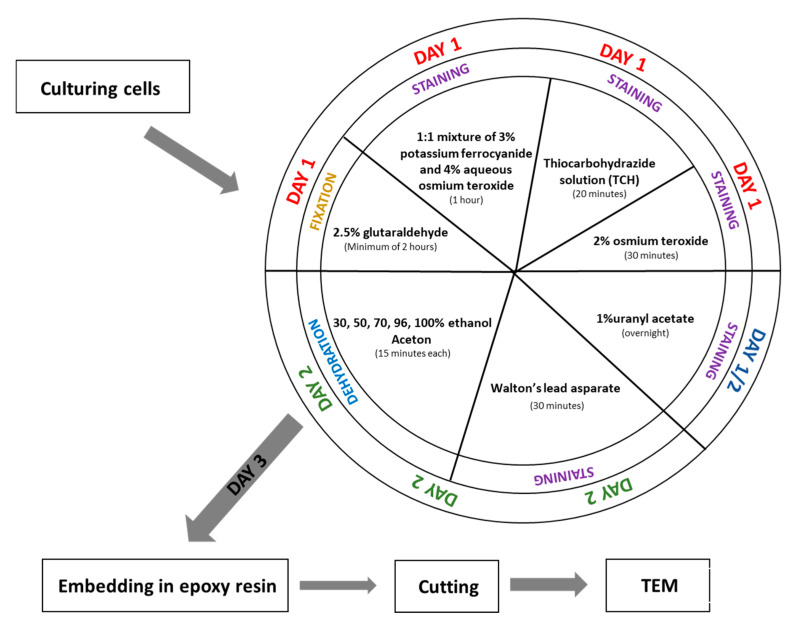
Scheme of procedure fixation for SBEM.

**Fixation and embedding (Figure 1)**

Day 1

1. Fix the cell pellet in 2.5% glutaraldehyde prepared in 0.1 M phosphate buffer (pH 7.4) at room temperature for a minimum of 2 h. (The fixation time can be extended if necessary.)
2. Wash the sample in 0.1 M phosphate buffer (pH 7.4) five times for 3 min each.
  (Note: If fixation time was extended, extend the washing steps accordingly.)
3. Incubate the sample in a 1:1 mixture of 3% potassium ferrocyanide (in 0.1 M phosphate buffer) and 4% aqueous osmium tetroxide for 1 h on ice.
4. While step 3 is ongoing, prepare the 1% thiocarbohydrazide (TCH) solution: Dissolve 0.1 g TCH in 10 mL of ddH_2_O. Incubate the solution at 60 °C for 1 h, swirling every 10 min to aid dissolution. Filter the solution through a 0.22 µm Millipore syringe filter. Note: TCH solution must always be freshly prepared before use.
5. After step 3, wash the sample in ddH_2_O five times for 3 min each.
6. Incubate the cell pellet in 1% TCH solution for 20 min at room temperature.
7. Wash the sample again in ddH_2_O five times for 3 min each.
8. Incubate the sample in 2% aqueous osmium tetroxide for 30 min at room temperature.
9. Wash the sample in ddH_2_O five times for 3 min each.
10. Add 1% aqueous uranyl acetate to the sample and incubate overnight at 4 °C (in the dark).

Day 2

1. Wash the sample in ddH_2_O five times for 3 min each at room temperature.
2. While washing, prepare En bloc Walton’s lead aspartate solution. First, make a 0.03 M aspartic acid solution; this acid will dissolve faster as the pH will be lower. En bloc Walton’s lead aspartate solution consists of 0.066 g of lead nitrate in 10 mL of a 0.03 M aspartic acid solution. Adjust pH to 5.5 with 1 N KOH. Incubate the mixture at 60 °C for 30 min. While in incubation, no precipitate should form.
3. Return to step 1. After incubation, wash the sample in ddH_2_O five times for 3 min each at room temperature.
4. Add to the cells pellet En bloc Walton’s lead aspartate solution and incubate at 60 °C for 30 min.
5. Wash the sample in ddH_2_O five times for 3 min each.
6. Dehydrate the cell pellet in a graded ethanol series for 15 min at each step: 30%, 50%, 70%, 80%, and 96% ethanol, followed by four changes of 100% ethanol (15 min each).
7. Incubate the sample for 15 min in a 1:1 solution of acetone and ethanol, then twice for 15 min in 100% acetone.
8. After dehydration, transfer the samples to a 50% epoxy embedding medium in acetone and incubate for 2 h. Then, place the sample in an incubator at 56 °C overnight to allow the acetone to evaporate.

**Transmission Electron Microscopy (TEM)**

Ultra-thin sections (70 nm thick) were cut using a Leica EM UC7 ultramicrotome. The sections were examined with a Hitachi H500 transmission electron microscope at 75 kV.

## 4. Results

**Figure 2:**
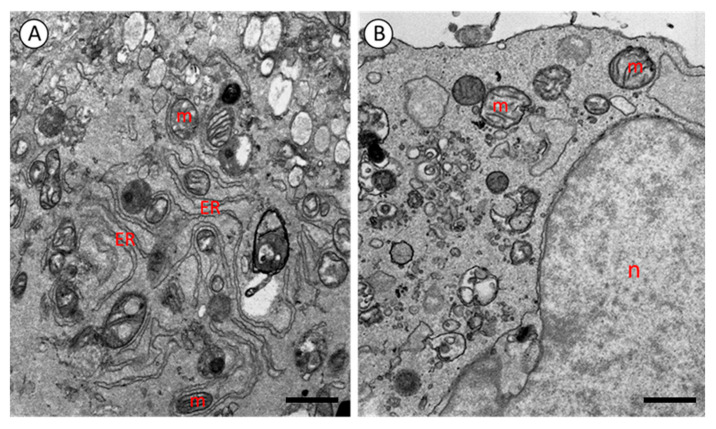
Electrograms of fibroblast (**A**) and iPS cell (**B**). m—mitochondria; ER—endoplasmic reticulum; n—nucleus. TEM. A: scale bar = 1.10 μm, B: scale bar = 1.09 μm.

Figure 2 presents representative transmission electron microscopy (TEM) images of induced pluripotent stem cells (iPSCs) and human dermal fibroblasts. The application of the optimized sample preparation and fixation protocol described above yielded satisfactory ultrastructural results for both cell types (Figure 2). No significant differences were observed between the iPSCs and fibroblasts in terms of fixation quality or image contrast, indicating that the protocol is equally effective for various cell types without requiring further modifications.

TEM analysis revealed excellent preservation of cellular structures. All cellular components were clearly visible, and no fixation artifacts were detected. High contrast was observed in membranes and intracellular organelles, including the nucleus, endoplasmic reticulum (ER), and particularly mitochondria. In both iPSCs and fibroblasts, mitochondria exhibited well-developed and clearly visible cristae (Figure 2).

## 5. Discussion

**Figure 3:**
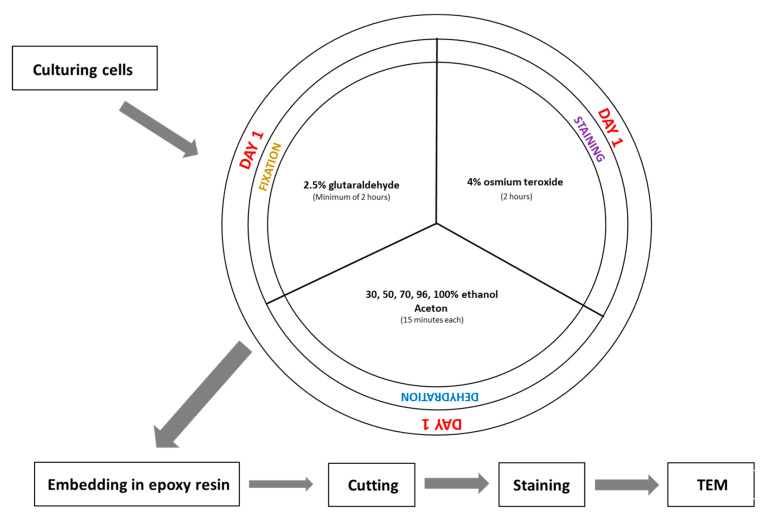
Scheme of traditional fixation for TEM.

Although SBEM is a type of electron microscopy, its fixation protocol differs significantly from that used in standard TEM imaging (Figure 1 and Figure 3). One of the main differences compared to TEM fixation is that the tissue is contrasted before its embedding. It helps to avoid contamination of TEM grids during contrasting materials after cutting. The SBEM preparation is more complex and time-consuming, due to the multiple staining and washing steps; however, this pre-embedding contrast enhances final imaging quality and eliminates the need for post-section staining, commonly required in TEM.

The foundational protocol for biological specimens preparation for SBEM was first described by Deerinck et al. in 2010 [14]. In our study, we applied and modified this protocol to enable high-resolution ultrastructural analysis of iPS cells generated via somatic cells’ reprogramming in comparison to somatic cells.

We introduced several changes to the original protocol, for example, we simplified the fixation process by using glutaraldehyde alone, omitting the mixture of glutaraldehyde, formaldehyde, and calcium chloride. We also substituted a cacodylate buffer with phosphate buffer throughout the entire procedure to minimize toxicity. These changes did not affect fixation quality, as confirmed by TEM imaging. The staining steps, like mixtures and time slots, are unchanged. Another optimization involved the dehydration process. We adjust the alcohol concentration and incubation durations based on our previous experience in electron microscopy sample preparation [15,16,17]. Our protocol closely resembles the one published by Śliwińska et al. 2021 [4] for brain tissue fixation. The differences are minimal, suggesting that this protocol may be broadly applicable to both tissue samples and cultured cells.

Unlike the approach described by Antao et al. [5,6], who fixed cells in a monolayer directly on culture plates, we detached the cells using 0.5 M EDTA (pH 8.0) before fixation. We also implemented slight changes in incubation times and reagent dilutions. Despite these procedural differences, the final samples exhibited high-quality fixation and excellent contrast, validating the robustness and versatility of our protocol.

Taken together, these findings demonstrate that our optimized protocol is both reliable and adaptable for ultrastructural analysis of diverse cell types.

## 6. Conclusions

The modified SBEM protocol presented here is a safe, efficient, and broadly applicable method for the ultrastructural analysis of in vitro cultured human cells. By implementing specific adaptations—including buffer substitution, simplified fixation, and optimized staining and dehydration—we achieved reproducible, high-quality results for both somatic and pluripotent cell types. This protocol enhances the methodological repertoire for 3D electron microscopy in biomedical research. Future applications may include its extension to 3D culture models such as spheroids.

## Data Availability

The data supporting the findings of this study, including representative TEM images, are available from the corresponding author upon reasonable request.

## Abbreviations

The following abbreviations are used in this manuscript:

CLEM	correlative light and electron microscopy
iPSCs	induced pluripotent stem cells
SBEM	serial block face electron microscopy
SEM	scanning electron microscopy
STEM	scanning transmission electron microscopy
TEM	transmission electron microscopy
TCH	thiocarbohydrazide
